# Characterizing the Anisotropic Elastic Properties of Auxetic Structures by Impulse Excitation Technique Combined with Inverse Parameter Identification

**DOI:** 10.3390/ma19122479

**Published:** 2026-06-09

**Authors:** Julian Rech, Yuchen Leng, Stefan Reinholz, Christian Dresbach, Danka Katrakova-Krüger, Christoph Hartl

**Affiliations:** 1 Institute of Technology, Resource and Energy-Efficient Engineering, University of Applied Sciences Bonn-Rhein-Sieg, von-Liebig-Straße 20, 53359 Rheinbach, Germany; 2 Material Laboratory, Faculty of Computer Science and Engineering Science, TH Köln—University of Applied Sciences, Steinmüllerallee 1, 51643 Gummersbach, Germany; yuchen.leng@th-koeln.de (Y.L.); stefan.reinholz@th-koeln.de (S.R.); danka.katrakova-krueger@th-koeln.de (D.K.-K.); 3 Manufacturing Technology, Faculty of Automotive Systems and Production, TH Köln—University of Applied Sciences, Betzdorfer Straße 2, 50679 Cologne, Germany; christoph.hartl@th-koeln.de

**Keywords:** auxetic metamaterials, additive manufacturing, thermal and mechanical characterization, impulse excitation technique, anisotropic elastic properties, inverse parameter identification

## Abstract

Auxetic metamaterials exhibit unique mechanical behavior due to their negative Poisson’s ratio, but reliable determination of their effective elastic properties remains challenging. In this study, an experimental–numerical approach is proposed to characterize additively manufactured polylactic acid (PLA)-based auxetic sandwich structures. Material properties were first assessed using tensile testing, melt flow rate/volume rate (MFR/MVR) measurements, Fourier-transform infrared (FTIR) spectroscopy, differential scanning calorimetry (DSC), dilatometry, and nanoindentation, revealing stable mechanical behavior, good processability, and slight increases in crystallinity induced by the printing process. Impulse excitation technique (IET) measurements provided highly reproducible resonant frequencies, demonstrating a strong dependence on core geometry and orientation. However, classical ASTM-based evaluation yielded non-physical elastic properties, highlighting its limitations for architected metamaterials. Finite element modal analyses, combined with inverse parameter identification, enabled the determination of effective elastic properties using a transversely isotropic homogenized model. This approach significantly improved the agreement between experimental and numerical results. The findings revealed pronounced anisotropy and orientation-dependent auxetic behavior, including a negative Poisson’s ratio for specific configurations. The proposed methodology provides a suitable framework for the reliable characterization and design of complex metamaterials.

## 1. Introduction

Auxetic metamaterials represent a unique class of engineered materials characterized by a negative Poisson’s ratio, exhibiting lateral expansion under tensile loading and contraction under compression [1,2,3,4]. This counterintuitive mechanical behavior results in advantageous properties such as enhanced energy absorption, improved fracture resistance, and superior vibration and damping performance [5,6]. These deformation mechanisms open up innovative application prospects in a wide range of applications, including aerospace, biomedical devices, protective gear, and flexible electronics [7]. Recent advances in additive manufacturing (AM) have enabled the fabrication of complex auxetic architectures with high geometric fidelity, significantly expanding the design space and practical applicability of these metamaterials [8,9]. However, AM processes introduce microstructural variations, residual stresses, and anisotropies that complicate the direct characterization of mechanical properties [10].

Despite these advances, the accurate determination of effective elastic mechanical properties, including Young’s modulus and shear modulus, remains challenging for lattice-based metamaterials due to their geometric complexity, structural hierarchy, and inherent anisotropy [8]. Conventional quasi-static testing methods, such as tensile and compression tests, provide valuable insights at the material and structural levels but they are often limited by boundary conditions, gripping-induced stress concentrations, and premature local buckling. These factors can lead to unreliable or non-representative material parameters for architected structures [9]. Consequently, non-destructive dynamic testing techniques have gained increasing attention as a quick and sensitive alternative to probe elastic properties by measuring natural frequencies of vibration.

In this context, the Impulse Excitation Technique (IET) has emerged as a robust and efficient method for determining elastic properties by analyzing the natural resonance frequencies of freely vibrating specimens [11,12]. The method is standardized and widely used for homogeneous and isotropic materials, allowing the evaluation of Young’s modulus, shear modulus, and Poisson’s ratio with high repeatability and minimal experimental effort [11,13]. Nevertheless, the standard ASTM approach for evaluating IET data assumes isotropic and homogeneous materials, which leads to inaccurate or non-physical results when applied to architected metamaterials that exhibit strong anisotropy and complex boundary conditions [11]. Therefore, the direct application of classical IET formulations to auxetic metamaterials remains non-trivial. The complex microstructural topology of auxetic lattices strongly influences their dynamic response, leading to resonance behavior that deviates from that of bulk materials [6]. In particular, the relationship between measured resonant frequencies and effective macroscopic elastic properties is affected by lattice geometry, relative density, and deformation mechanisms specific to auxetic designs [8]. Other studies have addressed the extension of the IET for determining negative Poisson’s ratio through refined formulations of resonance frequency ratios in circular disks [14], as well as the experimental investigation of internal friction and density-dependent damping behavior in auxetic structures such as re-entrant and tetrachiral geometries [15]. However, the application of IET for a comprehensive characterization of auxetic materials remains insufficiently explored and, consequently, the validity and interpretation of IET-derived material parameters for auxetic metamaterials requires further systematic investigation.

Numerical simulation provides a powerful framework to address these challenges. Finite element analysis (FEA), especially modal analysis, enables the accurate prediction of resonant frequencies and mode shapes while explicitly accounting for detailed lattice geometry, material behavior, and boundary conditions [16,17]. This approach facilitates a deeper understanding of how structural design and anisotropy influence dynamic response—a phenomenon that is often difficult to capture through experimental methods alone. Furthermore, FEA facilitates parametric studies to systematically investigate the sensitivity of effective elastic properties to changes in geometry, material anisotropy, and manufacturing defects. By combining simulation with experimental data, inverse parameter identification techniques can be employed to iteratively adjust material property inputs until the simulated modal characteristics closely match the measured resonance frequencies. This synergy between numerical modeling and experimentation not only improves the accuracy of effective property determination but also facilitates the development of reliable, homogenized material models [18]. Mao et al. [19] developed a multi-level inverse parameter identification framework to determine homogenized anisotropic elastic constants of cellular materials. By minimizing the discrepancy between simulated and reference displacement fields, the method enabled accurate characterization of the effective mechanical behavior of lattice structures. These models can be used for continuum-scale simulations, thereby bridging the gap between microstructure and macroscopic performance. In addition, Brezas et al. [20] used electronic speckle pattern interferometry to experimentally identify vibration mode shapes and validate finite element models of a splash cymbal. The results highlighted the effectiveness of interferometric techniques for accurately characterizing complex vibrational behavior and evaluating numerical model fidelity.

The novelty of the present work lies in the combination of impulse excitation testing with inverse finite-element-based parameter identification to determine a complete set of effective elastic constants of additively manufactured auxetic sandwich structures. In contrast to previous studies, the proposed methodology accounts for manufacturing-induced material variations, structural anisotropy, orientation-dependent behavior, and the limitations of classical ASTM-based IET evaluation. Furthermore, the use of a sandwich model with a transversely isotropic homogenized material model for the sandwich core calibrated through experimental modal data provides physically consistent elastic properties that can be directly used in continuum-scale engineering simulations.

In the initial phase of this work, the thermal and mechanical properties of the AM-filament were investigated, alongside the examination of process-induced effects on the material’s characteristics caused by the printing process. The main objective of this study was to determine the effective elastic properties of 3D-printed auxetic metamaterials using the IET as a non-destructive evaluation method. Elastic parameters were derived from experimentally measured resonance frequencies. To ensure a reliable interpretation of IET results for complex auxetic geometries, numerical modal analyses were conducted to correlate the measured dynamic response with the underlying effective elastic constants. This experimental–numerical approach established a versatile methodology for characterizing the effective elastic properties of auxetic structures while accounting for geometric design parameters and manufacturing-induced variations.

## 2. Materials and Methods

In this section, the materials and methods used to characterize the materials and printed auxetic structures are described, followed by an explanation of the finite element models used for designing the IET experiments and parameter identification.

### 2.1. Filaments and Sample Analysis

PLA Plus (PLA+) filament with a diameter of 1.75 mm (Filamentworld, Neu-Ulm, Germany) was investigated in this study. A Funmat Pro 410 FDM printer (Intamsys Technology, Shanghai, China) was used for printing with a layer thickness of 0.2 mm, a nozzle temperature of 200 °C, a bed temperature of 60 °C, a printing speed of 40 mm/s and an infill density of 100%. These settings were optimized to avoid defects according to the manufacturer’s guidelines. Five specimens per test were produced.

#### Methods for Filament Testing and Influence of Printing Process

Uniaxial tensile tests of the filament were conducted according to DIN 53504 [21] using a Zwick Roell Z100 TEW (ZwickRoell, Ulm, Germany), applying a load cell with a maximum measuring force of 2.5 kN and tensile speed of 120 mm/min. Melt flow rate (MFR) and melt volume rate (MVR) measurements of the filament material were conducted according to DIN EN 1133-1 [22] using a Zwick Roell Mflow device (ZwickRoell, Ulm, Germany) at 190 °C under a 5 kg load. The chemical structure of the filament material and 3D-printed sample 1 was analyzed by Fourier-transformed infrared spectroscopy (FTIR) with a Thermo Scientific Nicolet Nexus 470 spectrometer (Thermo Fisher Scientific/Nicolet, Madison, WI, USA). The number of scans (i.e., the number of measurements) was 32, with a wavelength range of 4000 to 500 cm^−1^. Thermal properties were assessed using differential scanning calorimetry (DSC) on a Netzsch DSC200PC system (NETZSCH Group, Selb, Germany). The first and second heating cycle range both from −10 to 250 °C with 70 mL/min nitrogen gas flow. The heating and cooling rate is 10 K per minute, and the capture rate is 300 Hz.

**Figure 1:**
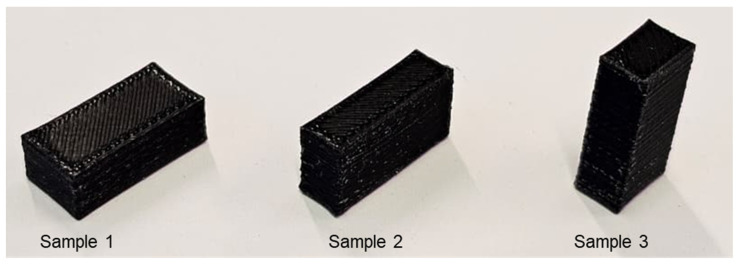
Illustration of sample 1, 2, and 3 with the bottom surface serving as the printing base.

For dimensional stability evaluation, dilatometry (DLM) measurements were performed on sections using a NETZSCH DIL 402C dilatometer (NETZSCH Group, Selb, Germany). Samples were heated up to 120 °C at a rate of 3 K/min under a nitrogen atmosphere flowing at 30 mL/min. A rectangular prism with dimensions of 15 × 8 × 5 mm^3^ is printed in three different build orientations, see Figure 1. Force-controlled nanoindentation tests were carried out on the three samples of the bottom and side surfaces using the LNP nano touch system (Ludwig Nano Präzision GmbH, Northeim, Germany), applying a maximum force of 1 N and a rounded indenter tip with a radius of 0.1 mm. These combined methods provided a comprehensive characterization of the mechanical, thermal, and structural properties of the initial material.

### 2.2. Additively Manufactured Auxetic Structures

Additively manufactured PLA-based sandwich concepts containing honeycomb or auxetic Star-4 core structures were investigated with a primary focus on mechanical characterization using the IET. Subsequently, reevaluation by finite element modeling was performed to determine the macroscopic material properties of the core structures.

#### AM Process

**Figure 2:**
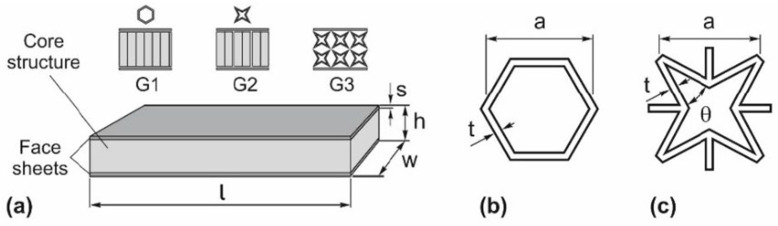
Concept of AM sandwich structures for experimental impulse excitation testing: (**a**) sketch, (**b**) honeycomb cell dimensions, and (**c**) Star-4 cell dimensions [23].

The information regarding the geometry of the core structure of the individual samples in this paper largely follows that of the previous work, for detailed information please refer to [23]. This study investigated the additive manufacturing of the sandwich structures depicted in Figure 2a, which have nominal dimensions of *l* = 123 mm, *w* = 60 mm, and *h* = 12 mm. The face sheets, which were printed with the core, had a thickness of 1 mm. Three core variants were examined: a conventional honeycomb structure (G1) with a cell size of 5.6 mm and a wall thickness of 0.58 mm (Figure 2b); an auxetic Star-4 structure with a cell size of 4.9 mm and a wall thickness of 0.42 mm (Figure 2c) tested in two orientations (G2, G3). The build direction, in which the individual layers were deposited in the additive manufacturing of the geometries, was along the height direction (h) for the G1 and G2 structures and face sheets, and along the width direction (w) for the G3 structure. The honeycomb structure was chosen as a practical benchmark due to its widespread use in industries like aerospace. The auxetic design was selected due to its tunable Poisson’s ratio via hinge stiffness, which offers versatile mechanical behavior. This must be considered in terms of the face sheets’ varying mechanical properties in the later evaluation. The respective dimensions of the sandwich structures and face sheets are summarized in Table 1.

### 2.3. Methods for Characterizing AM Auxetic Structures

Owing to the complex deformation mechanisms and non-classical effective behavior of auxetic metamaterials, the elastic evaluation approach of the impulse excitation technique according to ASTM E1876-22 [11] is not applicable. Instead, IET was employed solely to determine the resonant frequencies of the sandwich specimens. These experimentally obtained resonant frequencies represent a coupled response of the effective elastic properties and were subsequently used as target quantities in FEA simulations. By iteratively matching the numerical and experimental resonant frequencies, the macroscopic elastic properties of the auxetic structure were identified assuming a sandwich structure with transversely isotropic elastic behavior in the core.

#### 2.3.1. Impulse Excitation Technique

The impulse excitation experiments were conducted using a MK7 system (GrindoSonic, Leuven, Belgium) to measure specific resonant frequencies by manually exciting the corresponding vibration modes. Both the individual face sheets and the sandwich structures (G1–G3) were tested. For each vibration mode, five repetitions were performed at room temperature to ensure reproducibility. In conventional applications, the fundamental flexural, torsional, and longitudinal modes allow the determination of elastic stiffnesses according to ASTM E1876-22 [11]. However, sandwich structures—and particularly auxetic metamaterials—exhibit heterogeneous and anisotropic deformation mechanisms that violate the assumptions of homogeneous isotropic elasticity underlying the standard. Consequently, a direct elastic evaluation is not applicable, and additional vibration modes must be considered to capture the more complex mechanical response. Table 2 summarizes the specimen positioning, including impulse, detection, and support locations. The supports were placed at the nodal lines of the respective modes to ensure free, undisturbed vibrations and a physically meaningful determination of the resonant frequencies.

#### 2.3.2. Finite Element Modeling and Modal Analysis of Detailed Core Geometries

To capture the dynamic response of sandwich structures during impulse excitation, a 3D solid model with a quadratic shape function considering the specimen’s dimensions was introduced to the modal analysis of the FEA software ANSYS Workbench 2025R2. The face sheets were modeled as homogeneous PLA, while detailed geometric structures of the core G1, G2, and G3 were implemented to capture the actual deformation mechanisms of the metamaterials (Table 3). Material properties were taken from [25]. Free–free boundary conditions were applied to replicate the experimental IET setup. Mesh convergence studies were performed using the program-controlled mesh refinement to ensure numerical stability of the predicted resonant frequencies, and the optimal mesh size was determined to be 1.25 mm. For G1, the number of total elements and nodes was 103,164 and 399,155, respectively, with a CPU runtime of 216 s. For G2, the number of total elements and nodes was 323,261 and 526,984, respectively, with a CPU runtime of 293 s. For G3, the number of total elements and nodes was 116,640 and 622,709, respectively, with a CPU runtime of 262 s.

Although the detailed geometric core models provided insight into the complex deformation mechanisms and frequency response of the architected metamaterials, they do not directly yield transferable macroscopic elastic properties. The elastic behavior is governed by a strong coupling between local cell deformation and global vibration modes, which prevents a straightforward extraction of effective material parameters.

#### 2.3.3. Inverse Parameter Identification Assuming a Transversely Isotropic Core

Additional static finite element simulations were performed on the isolated detailed core structures. Uniaxial tensile loading, torsional loading, and bending were applied along the principal directions under small-strain conditions to estimate effective Young’s moduli, shear moduli, and Poisson’s ratios. To address the limitations of the detailed core model, statically derived elastic parameters were used as initial values for a homogenized core material model. For determining effective elastic properties, a 3D finite element model was built using quadratic solid elements with a mesh size of 5 mm considering the sandwich structure of the printed samples with a homogenized core. The face sheets were assumed to be linear elastic, while the homogenized core was assumed to be transversely isotropic. The number of total elements and nodes was 1200 and 6227, respectively, with a CPU runtime of 4.6 s.

The parameters derived with the 3D detailed model served as the basis for the inverse finite element identification procedure. Prior to the optimization, a sensitivity analysis was performed to ensure that the measured resonant frequencies gain enough information for identifying the required elastic constants.

For the optimization in Ansys optiSLang, the adaptive response surface method (ARSM) with a D-optimal linear approximation (start range: 0.5) was used. The ARSM was performed iteratively, with 5 to 20 iterations permitted for convergence. In this approach, the elastic properties of the face sheets were kept constant and the relationship between the elastic parameters of the homogenized core material and the resulting resonant frequencies was approximated by an adaptively refined response surface constructed from successive finite element modal analyses. The experimentally measured resonant frequencies obtained from IET were linked to the numerically predicted resonant frequencies by minimizing a least-square error (LSE) objective function

(1) $$LSE=\sum_{i=1}^{n}{\left(f_{i}^{exp}-f_{i}^{FEA}\right)}^{2}$$

where $f_{i}^{exp}$ and $f_{i}^{FEA}$ denote the experimental and numerical resonant frequencies of mode *i*, respectively. For the transversely isotropic assumption, the parameter space was reduced to five independent elastic constants considering each core structure yielding the compliance matrix [*S*]

(2) $$\left[S\right]=\left[\begin{array}{cccccc} \frac{1}{E_{x}} & \frac{-\mu_{xy}}{E_{x}} & \frac{-\mu_{zx}}{E_{z}} & 0 & 0 & 0\\ \frac{-\mu_{xy}}{E_{x}} & \frac{1}{E_{x}} & \frac{-\mu_{zx}}{E_{z}} & 0 & 0 & 0\\ \frac{-\mu_{xz}}{E_{x}} & \frac{-\mu_{xz}}{E_{x}} & \frac{1}{E_{z}} & 0 & 0 & 0\\ 0 & 0 & 0 & \frac{1}{G_{zx}} & 0 & 0\\ 0 & 0 & 0 & 0 & \frac{1}{G_{zx}} & 0\\ 0 & 0 & 0 & 0 & 0 & \frac{2\left(1+\mu_{xy}\right)}{E_{x}} \end{array}\right]$$

where

(3) $$E_{x}=E_{y}$$

(4) $$G_{yz}=G_{zx}$$

(5) $$\frac{\mu_{xy}}{E_{x}}=\frac{\mu_{yx}}{E_{y}},\frac{\mu_{xz}}{E_{x}}=\frac{\mu_{yz}}{E_{y}}$$

(6) $$G_{xy}=\frac{E_{x}}{2(1+\mu_{xy})}$$

with Young’s modulus *E*, shear modulus *G*, and Poisson’s ratio *µ* [26]. For the face sheets, elastic properties were experimentally determined and used as fixed parameters.

To ensure physically consistent frequency matching, the target modes were explicitly tracked throughout the optimization process to account for potential mode switching. In addition, the Modal Assurance Criterion (MAC), implemented via the NHV Toolkit, was employed to quantify the correlation between experimental and numerical mode shapes. This ensured that the optimization was performed on corresponding vibration modes and that changes in the elastic parameters did not result in unintended mode reordering.

## 3. Results and Discussion

In this section, the experimental and numerical results are presented and discussed with regard to the mechanical behavior of the filament, the printed auxetic structures and the identified material parameters.

### 3.1. Filament Analysis

#### 3.1.1. Tensile Testing

**Figure 3:**
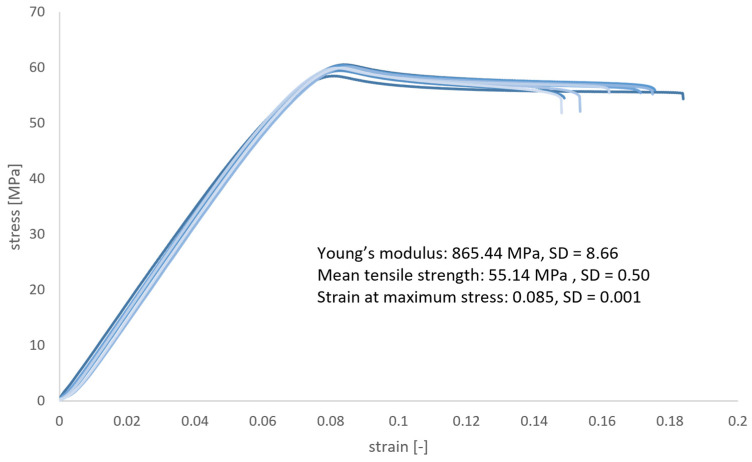
Engineering stress–strain curve of PLA+ filament with 10 repetitions.

The results of the tensile tests on PLA+ filaments are shown in Figure 3. Test results for the 10 samples showed minimal deviation, with an average Young’s modulus of 865.44 MPa, tensile strength of 55.14 MPa and an average strain under maximum stress of 0.085.

#### 3.1.2. Melt Flow and Melt Volume Rate Measurements

MFR and MVR measurements were taken on the PLA+ filament. The MFR is 25.59 ± 3.06 g/min and the MVR is 20.63 ± 2.46 cm^3^/10 min. Compared to the three materials studied in the previous paper [23], the PLA+ filament used in the present work exhibited higher MFR and MVR values. These results suggest that the filament has superior flowability, lower viscosity, and possibly a smaller molecular weight. This usually yields better print quality of the parts.

#### 3.1.3. FTIR Spectroscopy

**Figure 4:**
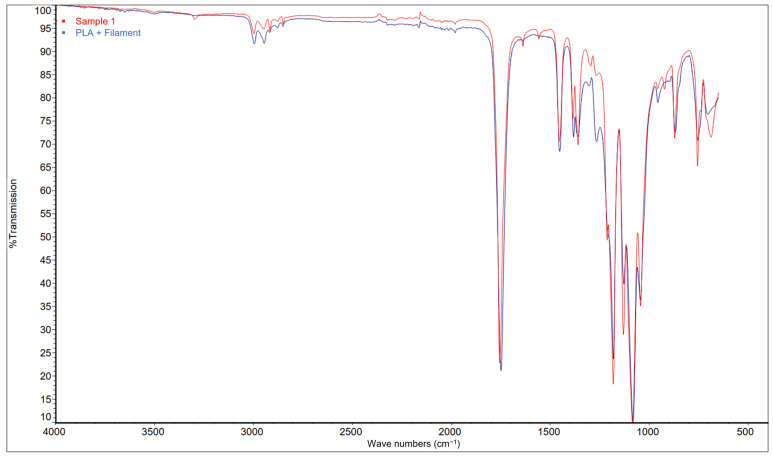
FTIR spectra of 3D-printed sample 1 and PLA+ Filament.

Figure 4 shows the FTIR spectra of the 3D-printed sample 1 and the original PLA+ filament material. Both spectra exhibit characteristic absorption bands of polylactic acid (PLA), confirming the material’s chemical integrity after printing. Specifically, there are prominent peaks that correspond to the stretching vibrations of the carbonyl group (C=O) at 1746 cm^−1^, and there is asymmetric and symmetric stretching of the methyl groups (CH_3_) at 2995 and 2946 cm^−1^, respectively. The C–O stretching vibration is also identified at 1080 cm^−1^. The bending vibrations of the methyl groups appear at 1452 cm^−1^ (asymmetric) and 1361 cm^−1^ (symmetric). The close similarity of the FTIR spectra of the printed sample and the original filament indicates that the PLA’s chemical structure remains largely unchanged during the 3D printing process. The FTIR spectra of the 3D-printed samples and the original filament reveal minor differences, suggesting that chemical and structural changes occurred during the printing process. The printed samples exhibit a more pronounced peak near 3296 cm^−1^, typically associated with O–H stretching vibrations. This suggests increased hygroscopicity or the formation of hydroxyl or amino groups, which is likely the result of heat exposure or surface oxidation during printing [27]. Additionally, the printed samples exhibit two distinct peaks in the 1650–1550 cm^−1^ wavenumber range, a weaker phenomenon observed in the original filament. These peaks may indicate the formation of new chemical functional groups, such as carboxylate, or structural rearrangements. These changes could be related to the shear deformation experienced by the filament during 3D printing. High shear forces may lead to H-O-H deformation vibration, and polymer chain breakage or induce structural rearrangement [28,29]. The original PLA filament exhibits lower FTIR peak intensities in this region compared to 3D-printed samples at about 1250. This region is associated with combination of the vibrations of CH and asymmetric C–O stretching vibration and typically indicates an increase in the crystallinity of the printed object [30]. Depending on the cooling rate, the 3D printing process typically increases the proportion of crystalline phases relative to the original amorphous material, thereby leading to higher peak intensities. These spectral differences suggest that the 3D printing process slightly alters the material’s internal chemical composition and molecular interactions, which may consequently affect its physical properties.

#### 3.1.4. Differential Scanning Calorimetry

**Figure 5:**
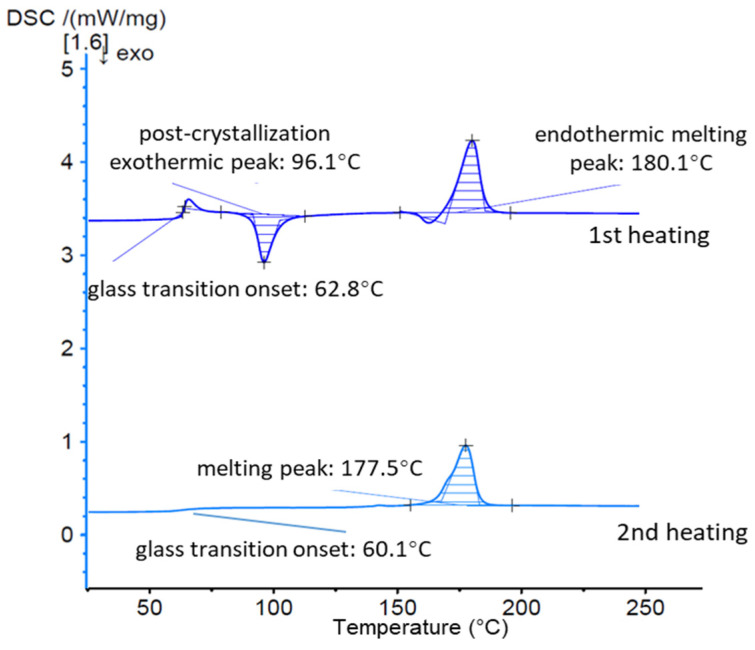
DSC thermograms of the PLA+ filament during first (dark blue) and second heating (light blue).

Figure 5 shows the DSC thermograms of the PLA+ filament during the first and second heating cycles up to 250 °C.

During the first heating cycle, the material exhibits a glass transition temperature (Tg) of approximately 64 °C. The enthalpy relaxation phenomenon occurs near the Tg and is typically caused by internal stresses, which are released as the amorphous regions soften. After the Tg, a distinct post-crystallization exothermic peak appears near 96 °C. This indicates that some amorphous polymer chains undergo additional cold crystallization as the temperature increases. Another smaller exothermic peak is visible right in front of the melting process at approx. 170 °C and is connected to the recrystallization of smaller imperfect crystals. The endothermic melting peak occurs around 180 °C, which corresponds to the melting of crystalline regions formed during manufacturing or prior cooling. This melting peak is characteristic of PLA and reflects the crystalline fraction in the filament [31]. The calculated crystallinity is approximately 39/93 = 42% (93 J/g is the literature value for a theoretically fully crystalline PLA [32]), indicating that this PLA material has a balanced structure—it is partially crystalline but still contains a substantial amount of amorphous material.

During the second heating cycle (lower curve) no exothermic post-crystallization was observed, indicating that the cooling rate was sufficiently slow to allow the material to fully crystallize. Consequently, the glass transition temperature during the second heating cycle was not clearly defined, with an initial value of 60.1 °C. However, the melting peak shifts slightly to approximately 175 °C, suggesting subtle differences in the crystalline structure or perfection due to the polymer’s prior thermal history.

#### 3.1.5. Dilatometer Measurements

**Figure 6:**
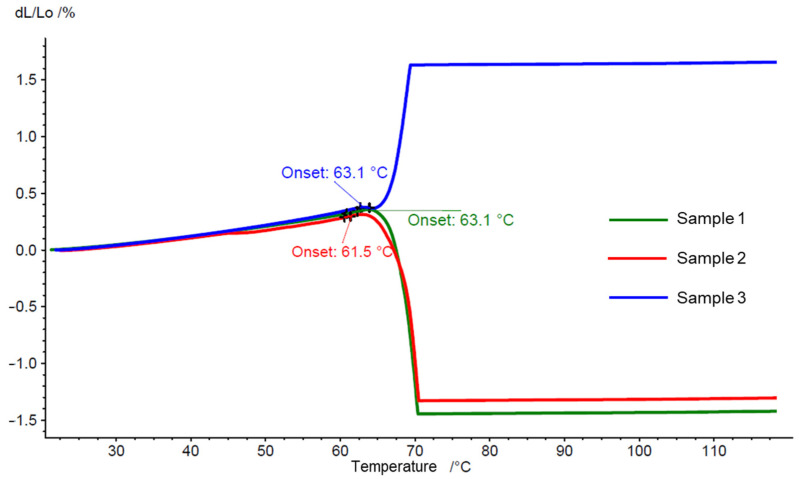
Dilatometer measurements of printed structures.

The DLM of the printed geometries, Figure 1, have shown that the different print directions exhibited similar behavior up to the glass transition temperature but significant differences in the progress of the recorded curves above this temperature, as shown in Figure 6. This behavior is attributed to the increased mobility of polymer chains and the relaxation of oriented chains within the filament bundles above the glass transition temperature. After passing the glass transition temperature, the ratio of the change in length (d*L*) to the initial length (*L*_0_) decreased for samples 1 and 2, while sample 3’s elongation increased. Thermal shrinkage, which is caused by orientation relaxation, impacted the dimensional stability and uniform thermal expansion of the printed parts.

Additionally, the figure illustrates the onset of thermal expansion, which is closely related to the beginning of the glass transition temperature (about 62.8 °C) comparable to the DSC results for the filament. Below this temperature, the material exhibits nearly linear expansion. Beyond this point, the slope of the curve increases abruptly due to an increase in molecular chain mobility.

#### 3.1.6. Nanoindentation

**Figure 7:**
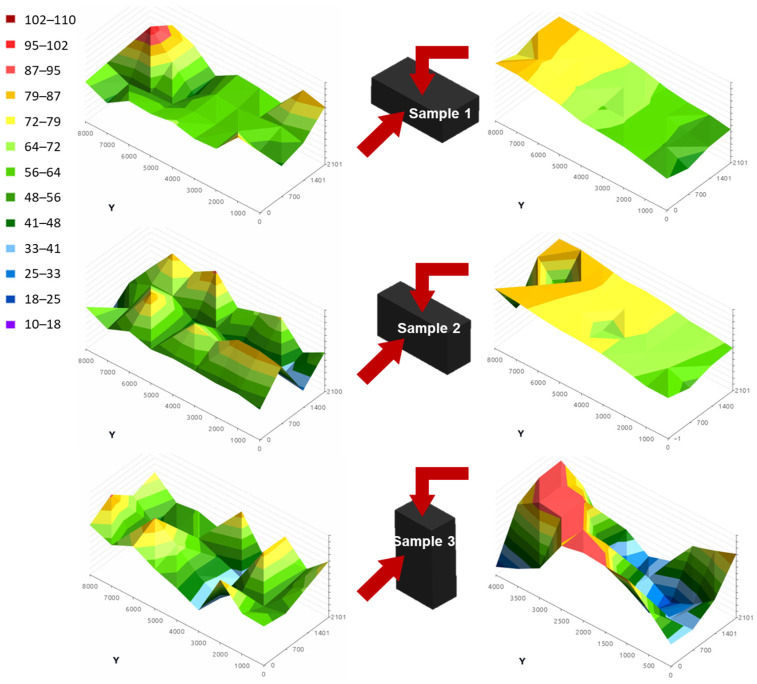
Visualization of measurement locations and generated nanoindentation 3D stiffness mapping of side and top sides of three samples (unit: mN/µm).

Figure 7 shows the surface measurement areas on the printed samples, along with the corresponding 3D stiffness distribution maps from nanoindentation. The measurements were conducted on the top and side surfaces of each of the three printed samples, with 36 detected measuring points, as shown in the middle of Figure 7. The stiffness maps on the left are from the left sides of the samples, while the maps on the right are from the top sides, showing different spatial variations in stiffness across the measured surfaces. Figure 7 illustrates the surface measurements of the samples and nanoindentation 3D mapping. The top and sides of the filament and the three samples were measured separately. Due to surface roughness and irregularities caused by the boundaries between printed layers, significant variations in the mapping results are evident, especially on the sides of the samples and the top surface of sample 3. The filament has slightly higher average stiffness than the printed structures, but the stiffness of the samples differs minimally; sample 3 is slightly less stiff than the other two.

**Figure 8:**
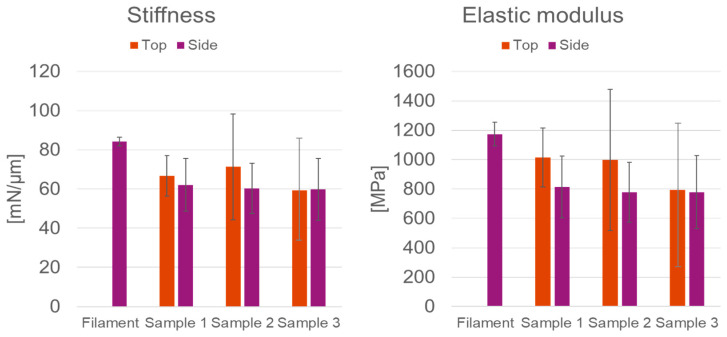
Comparison of stiffness and elastic modulus obtained by nanoindentation tests on filaments and the three samples.

The stiffness and elastic modulus values shown in Figure 8 were obtained from surface mapping based on 36 single-point indentation measurements. During the unloading phase following indentation, the contact stiffness is determined by the slope of the force-indentation depth curve. The elastic modulus is then calculated based on this stiffness and the contact area. The average elastic modulus of the filament is 1173 MPa, which is higher than the 866 MPa obtained from uniaxial tensile testing. This is partly related to surface strengthening; due to the manufacturing process of the fibers (extrusion), the polymer chains at the surface may be more strongly oriented or cool more rapidly, thereby forming a “skin” that is stiffer than the core. On the other hand, plasticity plays a role. In the classical analysis using the Oliver–Pharr method, the modulus is calculated based on the unloading curve. For polymers, plastic deformation near the indenter tip can artificially inflate the calculated modulus by as much as 60% [33]. However, the average elastic modulus of the 3D-printed samples is 862 MPa, and the data exhibit significant deviation. Micro-pores (voids) inevitably form between print layers during the 3D printing process. Since the elastic modulus depends directly on density, this porosity reduces the effective modulus of the overall structure compared to solid filament [34]. This results in significant deviations in the data. Furthermore, 3D-printed surfaces are rough by nature, meaning even the slightest error can lead to substantial fluctuations in the results.

### 3.2. AM Auxetic Structures

#### 3.2.1. Impulse Excitation Technique-Experiments

As described in Section 2 for the experimental part, Table 4 summarizes the determined resonant frequencies of the sandwich structures (G1–G3) and the isolated face sheets obtained via impulse excitation. The results demonstrate a high repeatability for all vibration modes, with standard deviations (SD) < 0.3% for the sandwich specimens. This confirms that the IET measurements provide a robust and reliable dataset suitable for subsequent inverse identification of mechanical properties.

A pronounced dependency of the resonant frequencies on the core architecture and orientation is observed. For the fundamental flexural mode (Mode 1), G1 exhibits the highest frequency (1213 Hz), followed by G2 (1085 Hz), whereas G3 shows a reduced frequency of only 699 Hz. Similar trends are observed for torsional (Mode 2) and higher-order bending modes. This behavior indicates substantial differences in global bending and torsional stiffness induced by the auxetic core topology and its orientation relative to the specimen axes.

The longitudinal mode (Mode 3) shows comparatively smaller differences between G2 and G3, suggesting that axial stiffness is less sensitive to auxetic orientation than bending- or shear-dominated deformation modes. In contrast, higher modes (Modes 4–6) exhibit increasingly strong deviations between the different sandwich designs, highlighting the complex coupling between local cell deformation mechanisms and global vibration behavior in auxetic metamaterials.

The resonant frequencies of the face sheets are much lower than those of the sandwich structures, reflecting their reduced thickness and bending stiffness. The differences between the face sheets of G1/G2 and G3 underline the influence of printing orientation on stiffness and has to be considered in the numerical modeling. Overall, Table 4 clearly demonstrates that the measured frequencies cannot be interpreted using classical homogeneous beam or plate assumptions, motivating the need for advanced numerical parameter identification approaches.

Table 5 presents the mean elastic moduli and Poisson’s ratios calculated according to ASTM E1876-22 [11] using the experimentally measured resonant frequencies. The Young’s moduli derived from flexural vibration vary widely between the different geometries, ranging from approximately 430 MPa for G3 to over 1000 MPa for G1 and G2. The shear moduli obtained from torsional vibration are approx. 1/3 of the flexural modulus reflecting the same trend. Applying the iterative approach according to ASTM E1876-22 [11] yields the Poisson’s ratio *µ* with several values exceeding the theoretical upper bound of 0.5, while negative values are obtained from the longitudinal–torsional mode combination. Such results are non-physical for conventional elastic continua and demonstrate that the ASTM equations attempt to force complex, anisotropic structural behavior into an oversimplified constitutive framework.

Even for the face sheets the ASTM-derived moduli differ depending on vibration mode due to the build direction during AM. Note that for the G3 face sheets, two moduli were calculated due to the different build direction (horizontal: 2711 MPa, vertical: 2381 MPa). For the sandwich structures, the situation is substantially worse, as the dynamic response is governed by bending–stretching coupling, shear deformation of the core, and auxetic cell rotations. Consequently, Table 5 provides strong experimental evidence that classical IET-based elastic evaluation is unsuitable for auxetic sandwich structures and should be replaced by a numerical parameter identification.

The thermal, microstructural, and mechanical investigations revealed that the material properties of the printed components differ from the nominal filament properties due to processing-induced effects such as crystallization, molecular orientation, interlayer bonding, porosity, and surface irregularities. These observations highlight the inherent heterogeneity and anisotropy introduced by the AM process and demonstrate that the effective mechanical response of the printed auxetic structures cannot be reliably described using manufacturer-provided material data alone. Consequently, an inverse identification procedure based on experimentally measured modal characteristics was employed to determine homogenized effective material parameters that account for both the material transformations occurring during printing and the influence of the cellular architecture.

Furthermore, the observed scatter in nanoindentation and thermomechanical measurements provides experimental evidence for local material variability, which contributes to the discrepancies between idealized numerical models and the behavior of the specimens and therefore motivates the use of a model-updating framework based on experimental modal data.

#### 3.2.2. Modal Analysis of Detailed Sandwich Structures

The comparison of experimental IET resonant frequencies with numerical modal analyses reveals a hierarchy in the predictive capability of the investigated modeling approaches when expressed in terms of relative frequency differences (Table 6).

Introducing the detailed geometric representation of the core architecture with PLA properties taken from [25] produces low percentage differences relative to IET. For G1 and G2, the detailed core FEA reproduces the experimental frequencies with differences generally below 5% and 10%, respectively, for the fundamental flexural, torsional, and longitudinal modes. This similarity confirms that explicit modeling of cell-level deformation mechanisms, especially bending and rotational influences in the connection support between the Star-4 architectures, is essential for capturing the experimentally observed dynamic response. However, for the auxetic configuration G3, notable differences remain, with relative deviations of approximately 10–15% for the lower modes and up to about 45% for higher-order modes, indicating limitations of geometry-only modeling under isotropic material assumptions. Nevertheless, considering detailed cell kinematics separately provides information about effective properties which can be introduced to the optimization procedure of the anisotropic homogenized core model to determine global mechanical properties. The elastic properties identified from the static FEM simulations support the assumption of a transversely isotropic material model for the core structure (G1: *E*_X_ = *E*_Y_ ≈ 100 MPa, *E*_Z_ ≈ 625 MPa/G2: *E*_X_ = *E*_Y_ ≈ 100 MPa, *E*_Z_ ≈ 800 MPa/G3: *E*_X_ = *E*_Z_ ≈ 60 MPa, *E*_Y_ ≈ 800 MPa).

#### 3.2.3. Inverse Parameter Identification Assuming Transversely Isotropic Behavior

The transversely isotropic homogenized core model provides even lower and the most consistent percentage differences with respect to the IET results using the aforementioned optimization procedure. For G1, relative deviations are generally below 3.5% across all investigated modes, demonstrating an excellent match between experiment and simulation. For G2, the differences remain moderate, typically below 5% for bending- and torsion-dominated modes and below approx. 8% for the longitudinal mode. Even for the more complex auxetic configuration G3, the transversely isotropic model substantially reduces the discrepancies observed in the isotropic and detailed isotropic approaches, limiting the percentage differences to approximately 15–21% for the most sensitive modes while correctly reproducing the overall frequency hierarchy.

The transversely isotropic elastic properties, Table 7, identified for the core material, reflect the underlying auxetic deformation mechanisms. The reference structure G1 exhibits low in-plane stiffness (*E*_X_ = *E*_Y_ = 86 MPa) combined with a higher out-of-plane modulus (*E*_Z_ = 440 MPa) and moderate shear stiffness (*G*_XY_ = 32 MPa, *G*_XZ_ = *G*_YZ_ = 107 MPa), together with exclusively positive Poisson’s ratios (*µ*_XY_ = 0.323, *µ*_XZ_ = *µ*_YZ_ = 0.235), consistent with bending-dominated deformation of a conventional cellular core [35]. In contrast, the auxetic configuration G2 shows increased stiffness in all principal directions (*E*_X_ = *E*_Y_ = 154 MPa, *E*_Z_ = 915 MPa) and enhanced shear resistance (*G*_XY_ = 113 MPa, *G*_XZ_ = *G*_YZ_ = 64 MPa), accompanied by a near-zero in-plane Poisson’s ratio (*µ*_XY_ ≈ 0), indicating a transition toward auxetic kinematics. Although a more pronounced auxetic response for G2 in terms of a strongly negative in-plane Poisson’s ratio (*μ*_XY_ ≪ 0) might be expected based on the unit-cell topology, the identified near-zero value suggests that the auxetic deformation mechanisms are partially constrained. This behavior is attributed to the direct connection between the core and the face sheets, which restricts lateral cell rotation and limits the development of macroscopic auxetic coupling under global deformation. The most pronounced auxetic response is observed for G3, which exhibits strong directional anisotropy with high stiffness in the y-direction (*E*_Y_ = 800 MPa) and markedly lower stiffness in the orthogonal directions (*E*_X_ = *E*_Z_ = 29 MPa), very low shear moduli (*G*_XY_ = 12 MPa, *G*_XZ_ = *G*_YZ_ = 9.75 MPa), and a clearly negative Poisson’s ratio in the x-z plane (*µ*_XZ_ = −0.175). The remaining discrepancies observed for G3 can be attributed to the combined effects of manufacturing-induced imperfections and the limitations of representing a highly anisotropic auxetic architecture by a sandwich structure with a transversely isotropic homogenized material model for the core section. Similar challenges have been reported for lattice metamaterials, where local rotational deformation mechanisms, geometric imperfections, porosity, and material heterogeneity limit the achievable agreement between experiments and homogenized finite-element models [9,33,34]. Furthermore, MAC values above 0.9 are generally considered indicative of strong mode-shape correlation in experimental modal analysis, even when residual frequency deviations remain present due to modeling and measurement uncertainties [36].

Overall, the combined trends in Young’s moduli, shear moduli, and Poisson’s ratios confirm that the inverse parameter optimization yields a physically consistent macroscopic representation of orientation-dependent auxetic behavior using a transversely isotropic material model (Table 7).

## 4. Conclusions

An experimental–numerical framework was developed for the characterization of additively manufactured auxetic sandwich structures, combining IET with inverse parameter identification. Complementary analyses (tensile testing, MFR/MVR, FTIR, DSC, dilatometry, and nanoindentation) confirmed that the PLA+ filament exhibits stable mechanical properties (tensile strength: 55.14 MPa) and good processability. The additive manufacturing process slightly increased crystallinity while preserving chemical integrity, resulting in a semi-crystalline material with temperature-dependent dimensional behavior and local stiffness variations due to layer-wise fabrication.

IET measurements showed high repeatability and a strong dependence of resonant frequencies on core geometry and orientation. Conventional ASTM-based evaluations yielded non-physical elastic properties, demonstrating their inapplicability to auxetic metamaterials. In contrast, inverse parameter identification using a transversely isotropic homogenized model provided physically consistent elastic parameters with good agreement between experimental and numerical results.

Distinct differences in dynamic behavior among geometries (G1–G3), particularly in bending- and torsion-dominated modes, highlight the influence of auxetic deformation mechanisms and anisotropy, while axial stiffness remained less sensitive to orientation. Finite element models considering a detailed core architecture provided good accuracy for G1 and G2 but remained limited for highly anisotropic structures (G3). The inverse approach assuming transversely isotropic behavior for the core material reduced frequency deviations to below 3.5% (G1) and 5–8% (G2), with improved predictions for G3.

The identified elastic properties reveal clear structure–property relationships: conventional honeycombs show bending-dominated behavior with positive Poisson’s ratios, while auxetic structures exhibit increased stiffness and, for G3, a pronounced negative Poisson’s ratio with strong anisotropy. The partially constrained auxetic response of G2 indicates the important role of core–face sheet interactions and boundary conditions.

Overall, the experimental–numerical framework presented in this work provides a versatile and adaptable tool for the characterization of complex metamaterials. It overcomes the limitations of conventional testing approaches and enables the determination of physically meaningful, anisotropic elastic properties. The methodology is particularly suitable for the design and optimization of advanced lightweight structures where accurate knowledge of effective material parameters is essential. Future work should focus on extending this approach to rate-dependent behavior, nonlinear deformation regimes, and alternative material systems to further enhance its applicability in engineering design.

## Figures and Tables

**Table 1:** Specimen’s geometry and density either measured directly or recalculated from face sheet properties.

Dimension	Unit	Sandwich Structure			Face Sheets	
		G1	G2	G3	G1/G2	G3
length *l*	mm	123.1	123.9	123.3	79.6	79.9
width *w*	mm	63.1	59.2	59.9	24.7	25.0
height *h*	mm	12.0	11.9	11.9	0.9	1.0
mass *m*	g	42.8	45.7	46.6	2.1	2.2
effective sandwich density *ρ*	g/cm^3^	0.46	0.52	0.53	1.18	1.09
calculated core density *ρ*	g/cm^3^	0.32	0.39	0.40		

**Table 2:** Setup of IET measurements for the different vibration modes with the corresponding mode shapes of the FE simulations (adapted from [24]).

Mode	Scheme	FE Simulation of Resonant Frequencies
1-flexural	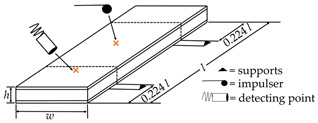	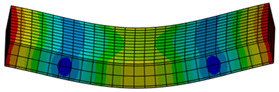
2-torsional	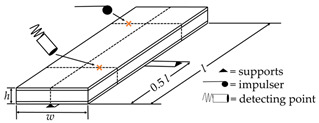	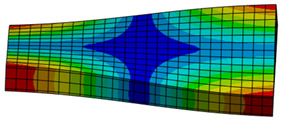
3-longitudinal	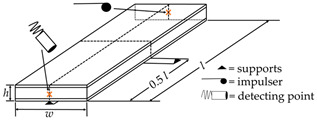	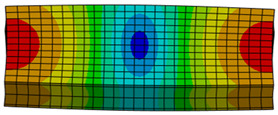
4	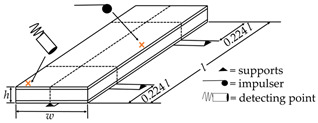	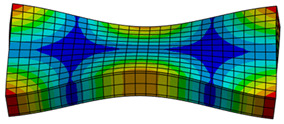
5	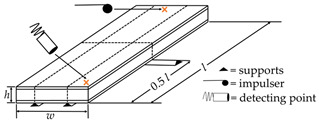	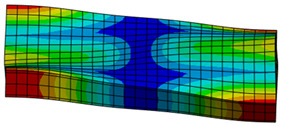
6	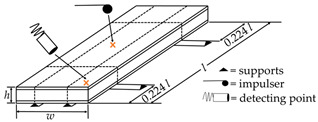	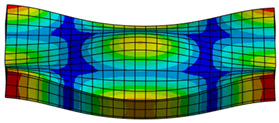

**Table 3:** Detailed isotropic FE-approach with coinciding mechanical properties for both face sheets and core.

Scheme		Mechanical Properties
G1	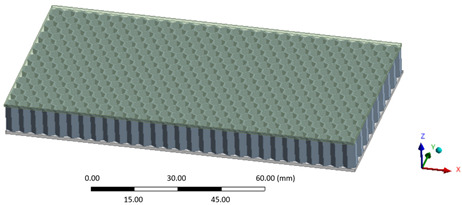	Face sheets material: PLA—1 mm *E* = 2400 MPa *ρ* = 1.245 g/cm^3^ *µ* = 0.33 Core material: PLA—10 mm *E* = 2400 MPa *ρ* = 1.245 g/cm^3^ *µ* = 0.33
G2	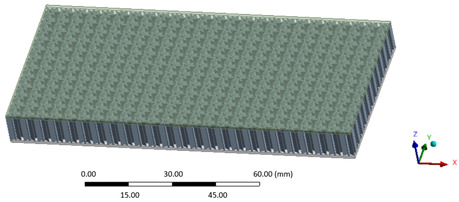	
G3	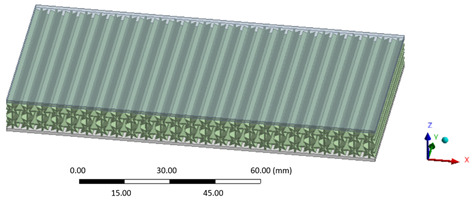	

**Table 4:** Experimental resonant frequencies of different vibration modes determined by impulse excitation for sandwich structures and face sheets.

Vibration Mode	Sandwich Structure						Face Sheets			
	G1		G2		G3		G1/G2		G3	
	Frequency *f* [Hz]									
	Mean	SD	Mean	SD	Mean	SD	Mean	SD	Mean	SD
1 (flex.)	1213	0.05	1085	0.12	699	0.21	227	0.26	251	0.13
2 (tors.)	1359	0.48	1231	0.70	695	0.47	449	0.68	453	0.80
3 (long.)	4396	2.39	3867	1.91	3928	1.96				
4	2847	5.03	2501	1.90	1413	1.02				
5	4761	2.00	3950	11.18	2752	4.50				
6	6126	5.39	5314	1.98	2827	5.29				

**Table 5:** Elastic moduli and Poisson’s ratios calculated according to ASTM E1876-22 [11] for sandwich and face sheet structures.

Vibration Mode	Sandwich Structure			Face Sheets	
	G1	G2	G3	G1/G2	G3
	Modulus *E*/*G* [MPa]				
1 (flex.)	1091	1037	430	2722	2711/2381
2 (tors.)	398	342	112	1066	900
3 (long.)	549	487	524	-	-
*µ* (flex./tors.)	0.37	>0.50	>0.50	-	-
*µ* (long./tors.)	−0.31	−0.29	>0.50	-	-

**Table 6:** FEA resonant frequencies of sandwich structures obtained by simulation (detailed core architecture with isotropic PLA-properties) or optimization (transversely isotropic core) compared to experimental IET resonant frequencies.

Vibration Mode	Sandwich (IET, Detailed Core, Transversely Isotropic Core)								
	G1			G2			G3		
	Frequency *f* [Hz]								
	IET	FEA^detail^	FEA^trans-iso^	IET	FEA^detail^	FEA^trans-iso^	IET	FEA^detail^	FEA^trans-iso^
1 (flex.)	1213	1149	1226	1085	1089	1138	699	623	848
2 (tors.)	1359	1234	1367	1231	1221	1258	695	628	804
3 (long.)	4396	4062	4414	3867	3953	4173	3928	3524	3698
4	2847	2569	2789	2501	2490	2479	1413	1292	1436
5	4761	4295	4844	4390	4468	4280	2752	4004	2330
6	6126	5481	5919	5314	5381	4958	2827	4125	2567

**Table 7:** Mechanical skin material properties used for and mechanical core material properties obtained from the ARSM optimization procedure for a transversely isotropic homogenized core approach.

Sandwich Structures				
Property	G1	G2	G3	Unit
face sheet				
*E*_X_	2720	2720	2710	MPa
*E*_Y_	2720	2720	2381	MPa
*E*_Z_	2720	2720	2381	MPa
*G*	1023	1023	900	MPa
*µ*	0.33	0.33	0.33	-
*ρ*	1175	1175	1092	kg/m^3^
transversely isotropic core				
*E*_X_	86	154	29	MPa
*E*_Y_	86	154	800	MPa
*E*_Z_	440	915	29	MPa
*G*_XY_	32	113	12	MPa
*G*_XZ_	107	64	9.75	MPa
*G*_YZ_	107	64	9.75	MPa
*µ*_XY_	0.323	0.000	0.181	-
*µ*_XZ_	0.235	0.202	−0.175	-
*µ*_YZ_	0.235	0.202	0.181	-
*ρ*	315	388	400	kg/m^3^
LSE	0.017	0.021	0.110	-
MAC objective function (f)	0.267	0.552	0.047	
MAC	0.990	0.995	0.902	-

## Data Availability

The original contributions presented in this study are included in the article. Further inquiries can be directed to the corresponding authors.
